# Novel H5 clade 2.3.4.6 viruses with both α-2,3 and α-2,6 receptor binding properties may pose a pandemic threat

**DOI:** 10.1186/s13567-014-0127-2

**Published:** 2014-12-17

**Authors:** Qunhui Li, Xuan Wang, Min Gu, Jie Zhu, Xiaoli Hao, Zhao Gao, Zhongtao Sun, Jiao Hu, Shunlin Hu, Xiaoquan Wang, Xiaowen Liu, Xiufan Liu

**Affiliations:** Animal Infectious Disease Laboratory, College of Veterinary Medicine, Yangzhou University, Yangzhou, Jiangsu 225009 China; Jiangsu Co-innovation Center for Prevention and Control of Important Animal Infectious Diseases and Zoonoses, Yangzhou, Jiangsu 225009 China

## Abstract

The emerging H5 clade 2.3.4.6 viruses of different NA subtypes have been detected in different domestic poultry in China. We evaluated the receptor binding property and transmissibility of four novel H5 clade 2.3.4.6 subtype highly pathogenic avian influenza viruses. The results show that these viruses bound to both avian-type (α-2,3) and human-type (α-2,6) receptors. Furthermore, we found that one of these viruses, GS/EC/1112/11, not only replicated but also transmitted efficiently in guinea pigs. Therefore, such novel H5 subtype viruses have the potential of a pandemic threat.

## Introduction, methods, and results

H5N1 subtype highly pathogenic avian influenza virus (HPAIV) was first isolated in sick geese in China in 1996,and has continued to evolve into over 10 distinct phylogenetic clades including different subclades based on the hemagglutinin (HA) gene [1]. Since 2010, H5 HPAIV subtypes which belong to the recommended novel clade 2.3.4.6 [2] with various neuraminidase (NA) subtypes (H5N1, H5N2, H5N6 and H5N8) have been detected in different domestic poultry in China [2-7]. Furthermore, the H5N8 virus-caused outbreaks have also been reported in wild birds and poultry in South Korea and Japan in January and April, 2014 respectively [8,9]. Here, we tested the receptor binding property of four novel clade 2.3.4.6 viruses, and guinea pigs were used as a mammalian model to examine the replication and transmission of these viruses. All animal experiments were approved by the Jiangsu Administrative Committee for Laboratory Animals (permission number SYXK-SU-2007-0005) and complied with the guidelines of the Jiangsu laboratory animal welfare and ethics of Jiangsu Administrative Committee of Laboratory Animals.

During surveillance of poultry for avian influenza viruses in live poultry markets in eastern China in 2013, one H5N8 avian influenza virus, A/duck/Shandong/Q1/2013 (DkQ1), was isolated from domestic ducks. The GenBank accession numbers for the DkQ1 segments are KM504098 to KM504105. Sequence analysis showed that all 8 genes of DkQ1 are closely related to those H5N8 viruses which have been reported in eastern China [4,5]. Furthermore, the HA gene of DkQ1 has high nucleotide identity with the H5N8 viruses circulating in South Korea and Japan in 2014 [8,9]. And all these H5N8 viruses belong to the recommended novel clade 2.3.4.6 [2]. In addition, one H5N8 virus A/duck/Jiangsu/k1203/2010 (Dkk1203) [4] and two H5N2 viruses A/duck/Eastern China/1111/2011 (DK/EC/1111/11) and A/goose/Eastern China/1112/2011 (GS/EC/1112/11) [3], which have been reported to circulate in eastern China, also possess HA genes belonging to the novel clade 2.3.4.6. Here, we investigated the receptor binding property and transmissibility of these four H5 (HPAIV) clade 2.3.4.6. All experiments with viruses were performed in a Biosafety Level 3 laboratory.

It is generally accepted that haemagglutinin-receptor-binding preference to α-2,6-linked sialylated glycans is the initial key step for a novel influenza-virus-causing pandemic [10]. First, we examined the receptor-binding specificity of these reassortant viruses by hemagglutination assays using goose red blood cells that were treated with a α-2,3-specific sialidase as previously described [11]. The A(H1N1)pdm2009 virus A/California/04/2009 (CA/04) and poultry H5N1 isolate A/mallard/Huadong/S/2005 (HD/05) [12] were used as controls. Theoretically, the sialidase digestion should abolish hemagglutination by α-2,3-specific viruses, whereas viruses that can bind to α-2,6-receptors should maintain hemagglutination activity with the treated red blood cells. The sialidase treatment did not affect the hemagglutination titer of CA/04, as shown in Table 1. Compared to untreated GRBC, these reassortant viruses still show some lower HA activity with α-2,3-sialidase-treated GRBC, which had only α-2,6-receptors (Table 1).

**Table 1:** **Hemagglutination titers of viruses from humans and animals** ^a^

**Virus stain**	**HA titers (log**_**2**_ **)**	
	**Untreated GRBCs**	**Treated GRBCs**
CA/04	6	6
HD/05	8	0
DK/EC/1111/11	8	7
GS/EC/1112/11	7	6
Dkk1203	7	5
DkQ1	7	6

^a^Hemagglutination titers were determined using goose red blood cells treated with α-2, 3-sialidas.

**Figure 1:**
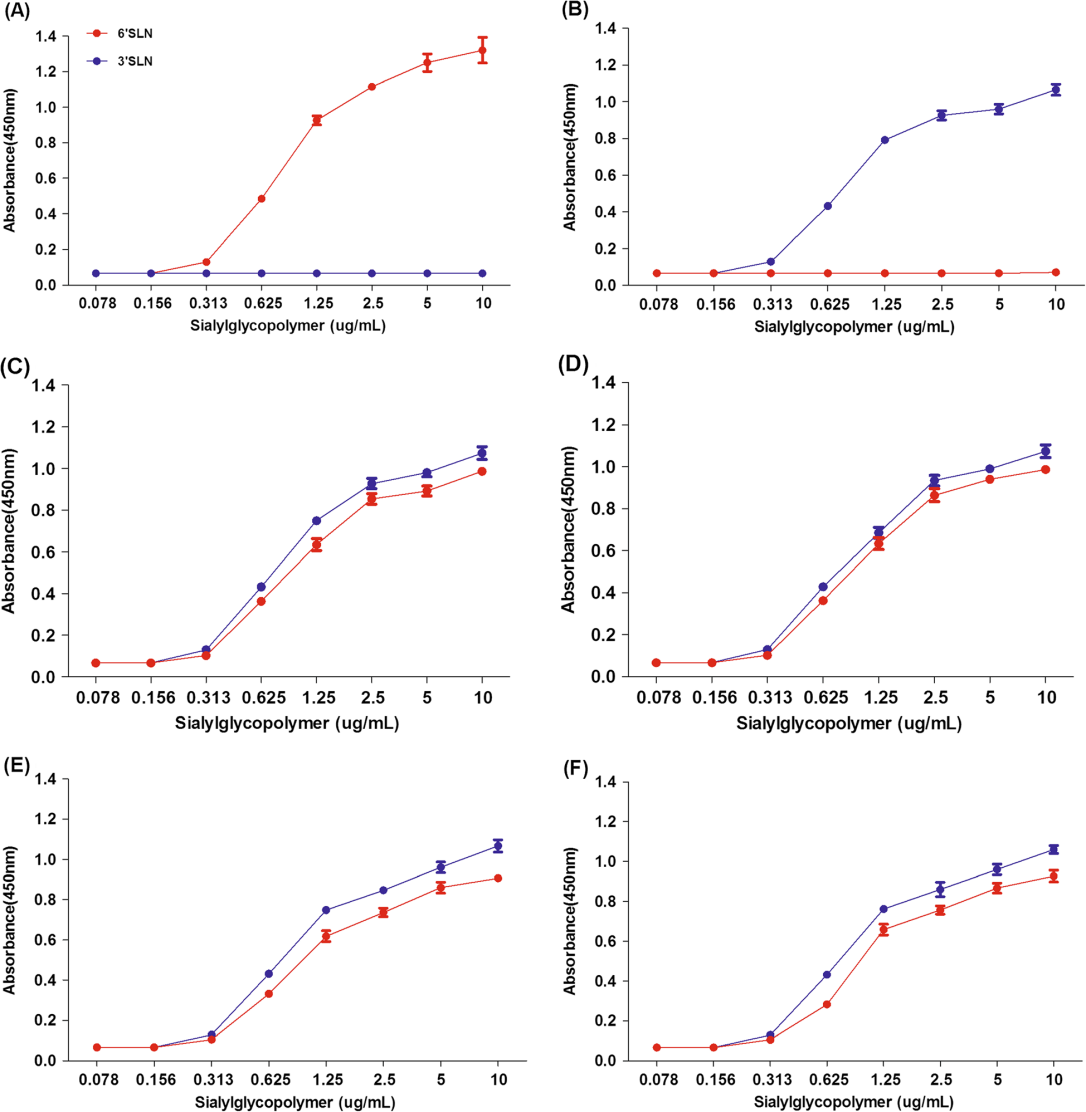
**Solid-phase receptor-binding assay of the H5 (HPAIV) clades 2.3.4.6.** Solid-phase receptor-binding assay of human isolate CA/04 **(A)**, poultry isolate HD/05 **(B)**, DK/EC/1111/11 virus **(C)**, GS/EC/1112/11 virus **(D)**, Dkk1203 virus **(E)** and DkQ1 virus **(F)**. Direct binding of viruses to sialylglycopolymers containing either 3’SLN-PAA or 6’SLN-PAA was measured. The data shown are representative of three independent binding experiments.

To characterize the receptor-binding properties of these viruses further, we performed solid-phase binding assays with different glycans as previously described [13]. Briefly, the synthetic sialylglycopolymers Neu5Aca2-3Galb1-4GlcNAcb (3’SLN)-PAA-biotin and Neu5Aca2-3Galb1-4GlcNAcb (6’SLN)-PAA-biotin (GlycoTech) were serially diluted in PBS and added to the wells of 96-well streptavidin coated microtiter plates (Pierce). The plates were blocked with PBS containing 2% skim milk powder, and 128 HA units of live virus was added per well. Chicken antiserum against the virus was diluted in PBS and added to each well. Bound antibody was detected by sequential addition of HRP-conjugated rabbit anti-chicken IgG antibody and tetramethylbenzidine substrate solution. The reaction was stopped with 1 M H_2_SO_4_, and the absorbance was read at 450 nm. Each sample was measured in triplicate. Our results show these reassortant viruses were bound to both avian-type (α-2,3) and human-type (α-2,6) receptors, whereas HD/05 and CA/04 viruses were preferentially bound to α-2,3 and α-2,6 receptors respectively, as expected (Figure 1). These results indicate that the HA of these reassortant viruses binds to α-2,3 receptors as well as to α-2,6 receptors.

To investigate the replication of these reassortant viruses, groups of four animals were anesthetized with pentobarbital natricum (40–50 mg/Kg) and inoculated intranasally with 10^6^EID_50_ of test virus in a 300 μL volume (150 μL per nostril). Two animals from each group were euthanized with CO2 on day 3 post inoculation (pi) and nasal washes, tracheas, lungs, kidneys, spleens, and brains were collected for virus titration in eggs. The remaining two animals were observed for two weeks for signs of disease and death. The A(H1N1)pdm2009 virus A/California/04/2009 (CA/04) and poultry H5N1 isolate A/mallard/Huadong/S/2005 (HD/05) were used as controls. As shown in Table 2, all reassortant viruses were detected in the nasal washes, tracheas and lungs of both inoculated animals, but only could be detected at lower titers in the trachea and lungs of infected guinea pigs. Virus was not detected in the brains, kidneys or spleens of any of the inoculated animals. We also infected two animals for each virus and observed them for two weeks for signs of pathogenicity. After two weeks pi, all of the animals seroconverted (Table 2). None of the animals showed disease signs during the observation period. These results indicate that replication of these reassortant viruses in guinea pigs is restricted to the respiratory system.

**Table 2:** **Virus replication and seroconversion in guinea pigs**

**Virus stain**	**Replication in guinea pigs** ^**a**^							**Seroconversion of the guinea pigs in transmission studies**	
	**Virus titers in organs(log**_**10**_ **EID**_**50**_ **/mL)**						**Seroconversion (positive/total)** ^**d**^	**Seroconversion: positive/total (HI titers)** ^**e**^	
	**Nasal wash** ^**b**^	**Lung**	**trachea**	**spleen**	**kidney**	**brain**		**Inoculated**	**Contact**
DK/EC/1111/11	4.8 ± 0.3	3.5 ± 0.2	1.2 ± 0.2	- ^c^	-	-	2/2	3/3(40,40,40)	0/3
GS/EC/1112/11	4.4 ± 0.7	3.2 ± 0.4	1.0 ± 0.1	-	-	-	2/2	3/3(80,40,40)	3/3(20,20,20)
Dkk1203	4.2 ± 1.6	2.8 ± 0.5	0.8 ± 0.1	-	-	-	2/2	3/3(80,80,40)	0/3
DkQ1	4.3 ± 1.3	2.7 ± 0.3	0.8 ± 0.2	-	-	-	2/2	3/3(80,40,40)	0/3
HD/05	-	1.0 ± 0.2	-	-	-	-	0/2		
CA/04	5.4 ± 0.4	4.5 ± 0.3	2.8 ± 0.4	-	-	-	2/2	3/3(160,320,320)	3/3(80,160,160)

^a^Groups of four guinea pigs were slightly anesthetized and intranasally inoculated with 10^6^EID_50_ of test virus in a 300 μL volume, 150 μL per nostril. Two animals from each group were euthanized on day 3 pi and samples, including nasal wash, lung, trachea, spleen, kidney and brain, were collected for virus titration in eggs. The remaining two animals were observed for two weeks and sera were collected at the end of the observation period.

^b^Data shown are log_10_EID_50_/mL.

^c^virus was not detected in the undiluted sample.

^d^Seroconversion was confirmed by hemagglutination inhibition (HI) assay.

^e^Sera were collected from guinea pigs on day 14 pi and treated overnight with Vibrio cholera receptor-destroying enzyme. Seroconversion was confirmed by hemagglutination inhibition (HI) assay.

**Figure 2:**
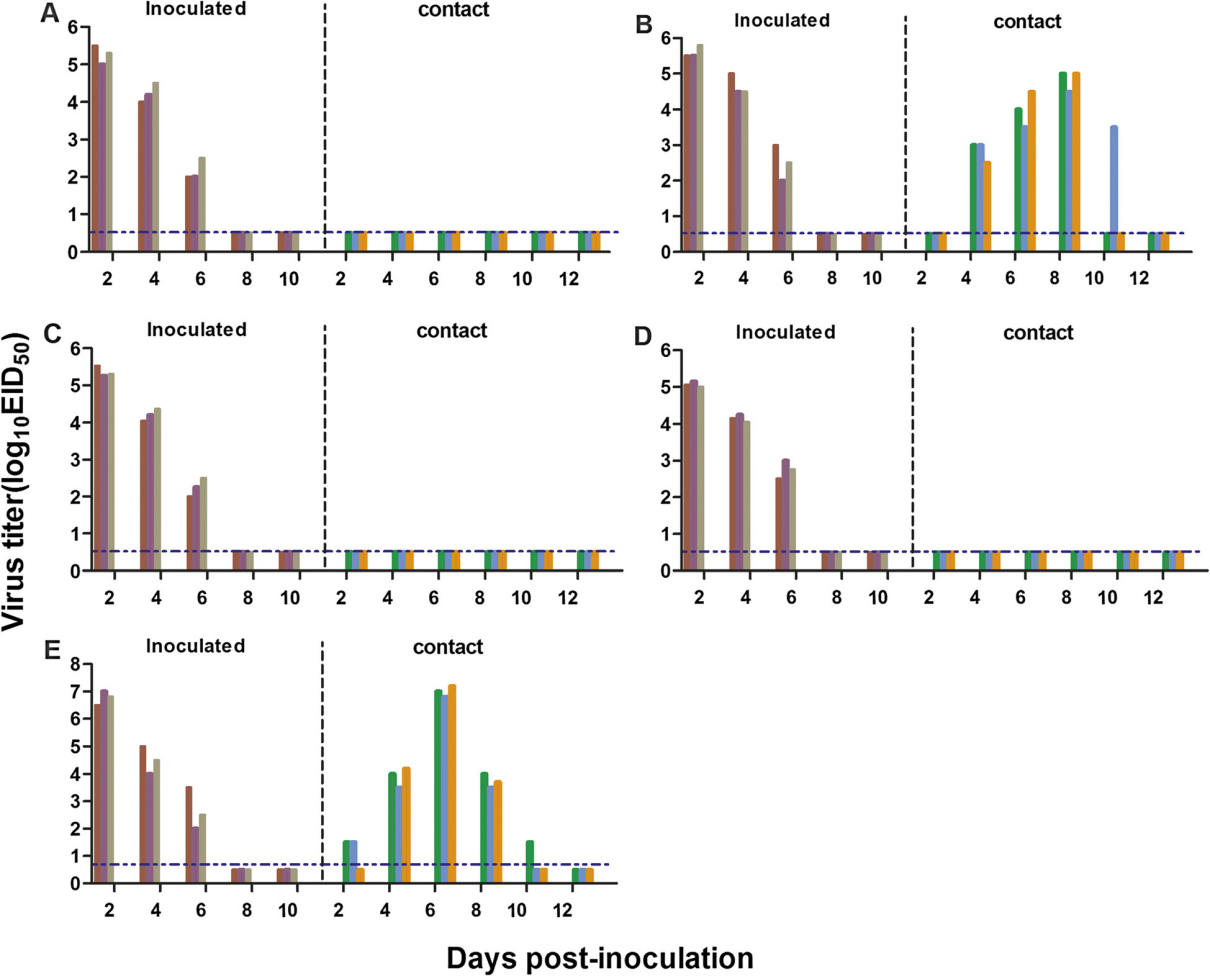
**Transmisson of the H5 (HPAIV) clades 2.3.4.6 in guinea pigs. (A)** DK/EC/1111/11 virus, **(B)** GS/EC/1112/11 virus, **(C)** Dkk1203 virus, **(D)** DkQ1 virus and **(E)** CA/04 virus. Each color bar represents the virus titer from an individual animal. The dashed blue lines in these panels indicate the lower limit of detection.

For the contact transmission studies, groups of three animals were inoculated intranasally with 10^6^EID_50_ of test virus and housed in a cage placed inside an isolator. Three naïve animals were introduced into the same cage 24 h later. Nasal washes were collected at 2 day intervals, beginning on day 2 pi (1 day post contact) and titrated in eggs. Sera were collected from guinea pigs at 14 days post inoculation (dpi) for hemagglutinin inhibition (HI) antibody detection [14]. Evidence of transmission was based on the detection of virus in the nasal wash and on seroconversion at the end of the two-week observation period. The A/California/04/2009 (CA/04) virus was used as controls. As shown in Figure 2, reassortant virus was detected in the nasal washes of all three inoculated guinea pigs between days 2–6 pi, but not in any of the contact guinea pigs. In the GS/EC/1112/11-inoculated groups (Figure 2B), virus was detected in the nasal washes of all three inoculated guinea pigs between days 2–6 pi, respectively and was also detected in the nasal washes of all three contact animals between days 4–8 pi. Seroconversion occurred in all inoculated groups (Table 2). In the contact animal groups, seroconversion was only observed among animals placed with the GS/EC/1112/11-inoculated animals. These results indicate that the transmissibility of the reassortant viruses in guinea pigs varies among viral strains, and of the four test viruses, only GS/EC/1112/11 transmit efficiently in this mammalian host.

## Discussion

Historically, changes in the receptor binding protein of influenza virus, HA, have been implicated in the initiation of a pandemic. It has been established for the H1N1 (1918), H2N2 (1957) and H3N2 (1968) pandemic viruses that a change in HA protein from a preference for α-2,3-linked sialic acids (avian receptor) to a preference for α-2,6-linked sialic acids (human receptor) is a prerequisite for efficient transmission of avian viruses to humans [10]. H5 HPAIV pose a serious pandemic threat due to their virulence and high mortality in humans, and their increasingly expanding host reservoir and significant ongoing evolution could enhance their human-to-human transmissibility. Recently, novel clade 2.3.4.6 H5 HPAIV with various NA subtypes (H5N1, H5N2, H5N6, and H5N8) were reported in Eastern China and South Korea [2-7,9,15]. Here, we evaluated their receptor specificity and transmission in guinea pigs. The results show that the viruses bound to both avian-type (α-2,3) and human-type (α-2,6) receptors. In humans, the α-2,6 receptor is expressed mainly in the upper airway, while the α-2,3 receptor is expressed in alveoli and the terminal bronchiole [16]. A virus with good affinity to both α-2,3 and α-2,6 receptors may especially be harmful, as it could infect efficiently via its binding to α-2,6 receptors in the upper airway and simultaneously cause severe infection in the lung via its binding to α-2,3 receptors. And this hypothesis is supported by the fact that one of the two well-characterized HA genes from the H1N1 1918 pandemic virus binds efficiently to both α-2,3 and α-2,6 receptors [17]. In addition, previous studies showed that the human-infecting novel H7N9 and the latest reassortant H10N8 avian influenza viruses yet have substantial affinity to both avian-type (α-2,3) and human-type (α-2,6) receptors [18,19]. Sequence analysis showed that novel H5 (HPAIV) clade 2.3.4.6 simultaneously carry a T160A mutation which results in the lack of an oligosaccharide side chain at 158–160 of HA, and it is critical for the H5 subtype influenza viruses tested to bind to human-like receptors and to transmit among a mammalian host [20,21]. Whether this T160A variation affects the receptor-binding property deserves further investigation. Previous studies showed that some H5 subtype influenza viruses can transmit efficiently in guinea pigs [21]. In this study, we also found that one of these viruses, GS/EC/1112/11, not only replicated but also transmitted efficiently in guinea pigs. These findings emphasize that continued circulation of these viruses may pose health threats for humans. Therefore, we need to intensify our effort to detect such viruses as early as possible.

## Abbreviations

HPAIV: Highly pathogenic avian influenza virus
HA: Hemagglutinin
NA: Neuraminidase
DkQ1: A/duck/Shandong/Q1/2013
Dkk1203: A/duck/Jiangsu/k1203/2010
DK/EC/1111/11: A/duck/Eastern China/1111/2011
GS/EC/1112/11: A/goose/Eastern China/1112/2011
CA/04: A/California/04/2009
HD/05: A/mallard/Huadong/S/2005
pi: Post inoculation
dpi: Days post inoculation
HI: Hemagglutinin inhibition
